# The Impact of Early Empirical Antibiotic Therapy on the Mortality of Bacteremia Patients with Klebsiella Infection: A Retrospective Cohort Study

**DOI:** 10.3390/jcm15010337

**Published:** 2026-01-02

**Authors:** Alaa Atamna, Tanya Babich, Amar Nahhas, Anan Zreik, Abed Agbaria, Shahd Dahamsheh, Mouhammad Haj Yahya, Haim Ben-Zvi, Jihad Bishara

**Affiliations:** 1 Infectious Diseases Unit, Rabin Medical Center, Beilinson Hospital, 39 Jabotinsky St., Petah-Tikva 49100, Israel; 2 Faculty of Medical and Health Sciences, Tel Aviv University, Tel Aviv 6139001, Israel; 3 Biostatistics Unit, Research Authority, Rabin Medical Center, Beilinson Hospital, Petah-Tikva 49100, Israel; 4 Beit Rivka Geriatric Medical Center, Petah-Tikva 49100, Israel; 5 Rabin Medical Center, Beilinson Hospital, Petah-Tikva 49100, Israel; 6 Clinical Microbiology Laboratory, Rabin Medical Center, Beilinson Hospital, Petah-Tikva 49100, Israel

**Keywords:** *Klebsiella* bacteremia, appropriate, early, mortality

## Abstract

**Background:** *Klebsiella* species are a leading cause of Gram-negative bacteremia associated with nosocomial infections. They exhibit higher antimicrobial resistance compared to other Enterobacterales, emphasizing their role as a “sentinel organism”. While the impact of inappropriate empiric therapy has been studied, data specific to *Klebsiella* bacteremia are limited due to small sample sizes. This study aims to provide high-resolution data on *Klebsiella* bacteremia and assess the impact of appropriate empirical therapy on clinical outcomes. **Methods:** We conducted a retrospective study of patients with *Klebsiella* bacteremia hospitalized at Beilinson Hospital between 2012 and 2022. Patients were categorized into two groups based on the appropriateness of empiric therapy. The primary outcome was 30-day all-cause mortality; subgroup analyses evaluated mortality in ESBL bacteremia treated with either carbapenems or piperacillin-tazobactam, and carbapenems versus aminoglycosides. Propensity score weighting and inverse probability treatment-weighted models were used to adjust for confounding. **Results:** Among 1132 patients, 79% received appropriate empirical therapy. This therapy was associated with reduced 30-day mortality (OR = 0.59, 95% CI: 0.46–0.76) and a shorter hospital stay (median 7 vs. 11 days, *p* < 0.001). Other significant risk factors for mortality included a higher Charlson comorbidity score (OR = 1.06), assistance with ADL (OR = 2.16), prior hospitalization (OR = 1.31), and a higher SOFA score (OR = 1.32). No significant mortality differences were observed in ESBL subgroups treated with carbapenems versus piperacillin-tazobactam (*p* = 0.2) or carbapenems versus aminoglycosides (*p* = 0.9). **Conclusions:** Early appropriate empirical therapy significantly reduces 30-day mortality in *Klebsiella* bacteremia. These findings highlight the importance of timely, appropriate empirical therapy and suggest choosing less broad-spectrum therapy. However, the lack of molecular data on resistance mechanisms limits the ability to assess strain-specific outcomes and may affect generalizability. Despite this, the study offers valuable insights for optimizing empirical therapy and advancing antimicrobial stewardship in the era of rising resistance.

## 1. Introduction

*Klebsiella pneumoniae* is recognized as the second most common cause of Gram-negative bacteremia, following *Escherichia coli* [1,2]. It is primarily a nosocomial pathogen, with the biliary and genitourinary tracts serving as the most frequent primary sources of infection. The mortality rate associated with *K. pneumoniae* bacteremia ranges from 20% to 30%, with higher rates observed in older adults, hospital-acquired infections, and cases of primary bacteremia [3].

This high mortality is compounded by the global crisis of antimicrobial resistance (AMR), where approximately 1.3 million deaths were attributed to AMR pathogens in 2019 internationally [4]. Where any Gram-negative organism has the potential to harbor resistance genes, they are most prevalent in *E.coli* and *Klebsiella* species [4]. Compared to *E. coli*, *K. pneumoniae* exhibits higher resistance rates to third-generation cephalosporins and carbapenems. This makes *Klebsiella* an important “sentinel” organism in antimicrobial resistance surveillance programs [5].

While numerous studies have investigated the impact of inappropriate empiric antibiotic therapy on Gram-negative bacteremia overall, studies specifically focusing on *Klebsiella* species bacteremia have been challenged by methodological limitations. The findings from existing studies are often inconsistent, largely due to variations in patient populations (e.g., sepsis vs. severe sepsis/septic shock, with or without bacteremia), differences in microbiological characteristics across Gram-negative organisms, and the failure to adequately adjust for time-dependent confounding and bias.

For example, a prospective multicenter study from England found that inappropriate empiric antibiotic therapy was not associated with increased 30-day mortality in Gram-negative bacteremia [adjusted odds ratio 0.92; 95% CI (0.5–1.66)] [1]. In contrast, another retrospective study reported a significant impact of inappropriate empirical therapy, but only among patients with a high baseline risk of death [6]. In addition, the MERINO trial, which included patients with ceftriaxone-resistant *E. coli* and *K. pneumoniae* bacteremia, showed that definitive treatment with piperacillin-tazobactam compared with meropenem did not show a survival benefit. However, only two thirds of the patients had received appropriate empiric therapy, and approximately 15% of patients in the piperacillin-tazobactam arm received carbapenem as empiric therapy [7].

The aim of our study is to generate high-resolution data on *Klebsiella species* bacteremia using inverse propensity score weighting to enable a more precise analysis of treatment appropriateness and its correlation with clinical outcomes.

## 2. Methods

### 2.1. Study Population and Setting

This retrospective cohort study included all consecutive adult patients (aged >18 years) who were hospitalized at Beilinson Hospital between January 2012 and December 2022 with *Klebsiella* species bacteremia. Patients were categorized into two groups based on the appropriateness of empirical antibiotic therapy: those who received appropriate empirical therapy and those who received inappropriate empirical therapy. The primary outcome of the study was 30-day all-cause mortality. Secondary outcomes included the length of hospital stay and admission to the intensive care unit (ICU) within one week of bacteremia presentation.

A subgroup analysis was conducted on 167 patients with extended-spectrum beta-lactamase (ESBL) producing *Klebsiella* bacteremia who had received appropriate empirical monotherapy within the first 24 h of culture collection. In this subgroup, 30-day mortality was compared between patients treated empirically with carbapenem monotherapy and those treated with piperacillin-tazobactam monotherapy. In a separate analysis involving 221 patients, outcomes were compared between those treated empirically with carbapenem monotherapy and those who received empirical aminoglycoside monotherapy. Patients receiving combination empirical therapy were excluded from this analysis to allow for a direct comparison between specific drug classes. Variations in cohort sizes reflect the availability of complete treatment and outcome data for each respective analysis.

### 2.2. Definition

Appropriate empirical antibiotic treatment: Intravenous antibiotics given during the first 24 h from culture collection and to which the Klebsiella isolate was sensitive in vitro. Patients who did not receive antibiotics within 24 h of blood culture collection were considered to be receiving inappropriate therapy. Patients who received combination empirical treatment were excluded.

### 2.3. Microbiological Methods

Blood was inoculated into Bactec blood culture aerobic and anaerobic bottles (BD, Franklin Lakes, NJ, USA). Upon receipt in the clinical microbiology laboratory, all Bactec blood culture bottles were incubated in the Bactec FX incubator (BD, Franklin Lakes, NJ, USA) for up to 5 days. Once positive, blood culture bottles were aseptically inoculated into blood agar, MacConkey, and chocolate agar (HyLabs Rehovot, Rehovot, Israel) and incubated for 18 to 24 h at 37 °C and 5% CO_2_. A Gram-stain slide was also prepared and reported within 1 h of positivity. Blood cultures with Gram-negative organisms were tested by matrix-assisted laser desorption ionization–time of flight mass spectrometry (MALDI-TOF MS), either from broth or directly from the isolate. After 24 h of incubation of the positive blood culture plate, suspected colonies were noted. The organisms were identified by matrix-assisted laser desorption ionization time of flight mass spectrometry (MALDI-TOF MS) (Bruker Daltonics, Billerica, MA, USA). Antimicrobial susceptibility was interpreted according to the guidelines of the Clinical and Laboratory Standard Institute (CLSI, M45-A3).

### 2.4. Data Collection

Data were retrieved from patients’ medical records in Beilinson Hospital. Collected variables included demographics (age, gender, age-adjusted Charlson comorbidity score [8]), residency before admission, hospitalization within three months prior to the index admission, and surgery within 30 days prior to the index admission. Information on comorbidities was also collected, including diabetes mellitus, ischemic heart disease, congestive heart failure, prior stroke, chronic kidney failure, hemodialysis, solid tumors, and hematologic malignancies. Bacteremia-related factors included resistance phenotype (non-ESBL, ESBL, CRE), source of bacteremia, appropriateness of empiric antibiotic therapy, the SOFA score [9] at bacteremia identification, and laboratory markers (WBCs, albumin, and creatinine) at bacteremia identification. Mortality outcomes were collected at different time frames (7, 14, 30 days).

### 2.5. Statistical Analysis

We compared the study variables based on the appropriateness of the empirical antibiotic therapy (appropriate empirical treatment, study group and the inappropriate empirical treatment, control group), using bivariate and multivariable logistic regression models. Continuous variables were assessed using the *t*-test (for normally distributed variables) or the Mann–Whitney U test (non-normally distributed variables). Variables with multiple categories were assessed using Chi-square or Fisher’s exact test, as appropriate.

To minimize selection bias and time-dependent confounding (such as immortal time bias) inherent in observational studies investigating the effect of treatment timing, we employed propensity score weighting (PSW). The propensity score for receiving appropriate empirical antibiotic therapy was calculated using a multivariable logistic regression model that included all baseline characteristics and established risk factors for mortality (e.g., age-adjusted Charlson comorbidity score, SOFA score, septic shock, acquisition setting, source of infection, and ESBL resistance status). This score was used to calculate the inverse probability treatment weight (IPTW) for each patient. IPTW models were then used to create a pseudo-population where the measured confounders were balanced across the appropriate empirical antibiotic therapy and inappropriate empirical antibiotic therapy groups, allowing for a more robust estimation of the causal effect of appropriate empirical antibiotic therapy on 30-day mortality. Variables with a *p* value <0.1 in the univariate analysis were also included in the final multivariable IPTW model. Results are presented as adjusted odds ratios with 95% confidence intervals (CIs).

Data analysis was performed using SPSS software(g SAS 9.4 software).

The study was conducted following the STROBE (Strengthening the Reporting of Observational Studies in Epidemiology) guidelines for observational studies and approved by the Rabin Medical Center ethical committee on 6 November 2022, with approval number RMC-0643-22.

## 3. Results

### 3.1. Demographic and Clinical Characteristics

This study included 1132 patients with *Klebsiella* bacteremia, of whom 79% (898/1132) received appropriate empirical treatment, and 21% (234/1132) received inappropriate treatment. Baseline characteristics are presented in Table 1. The median age of patients with *Klebsiella* bacteremia was 71 years (IQR: 60–79), and the majority were men, 60% (675/1132). Approximately half of the patients had been hospitalized within three months prior to the index admission, 47% (534/1132), and half required assistance with activities of daily living (ADL), 50% (565/1132). Patients who received appropriate empirical antibiotic therapy were more likely to have arrived from home (82% (608/898) vs. 73% (132/182); *p* = 0.004) and were less likely to be hospitalized in surgical wards (23% (212/898) vs. 33% (77/234); *p* = 0.004). The median Charlson comorbidity index was similar in both groups, 5 (4–8) vs. 5 (4–9); *p* = 0.5. One-third of the patients had diabetes mellitus, 27% (306/1132), and one-fourth had a solid tumor, 25% (279/1132).

### 3.2. Bacteremia and Clinical Characteristics

*K. pneumonia* accounted for the vast majority of bacteremia cases, 87.6% (992/1132), followed by *K. oxytoca*, 6.2% (70/1132), and *K. aerogenes*, 6.2% (70/1132). Non-ESBL Klebsiella bacteremia comprised two-thirds of bacteremia cases, 59% (671/1132), while ESBL Klebsiella bacteremia comprised 37.1% (420/1132). The urinary tract was the most common source of bacteremia, 40.5% (458/1130), followed by primary bacteremia, 25.8% (291/1130), and abdominal/biliary tract source, 13.2% (149/1130). The median SOFA score at the bacteremia onset was 4 (2–6). Patients who received inappropriate empirical antibiotics therapy were more likely to be mechanically ventilated at bacteremia onset, 20% (47/234) vs. 11% (100/898), *p* < 0.001), have higher creatinine levels and lower albumin levels, 1.47 (0.98–2.52) vs. 1.26 (0.87–2.01), *p* = 0.02 and 2.95 (2.5–3.5) vs. 3.2 (2.7–3.5), *p* = 0.02, respectively. In addition, patients with ESBL bacteremia were more likely to receive inappropriate antibiotic therapy, 67.1% (157/234) vs. 29% (263/898), *p* < 0.001.

### 3.3. Clinical Outcomes in Patients with Klebsiella Bacteremia

**Figure 1:**
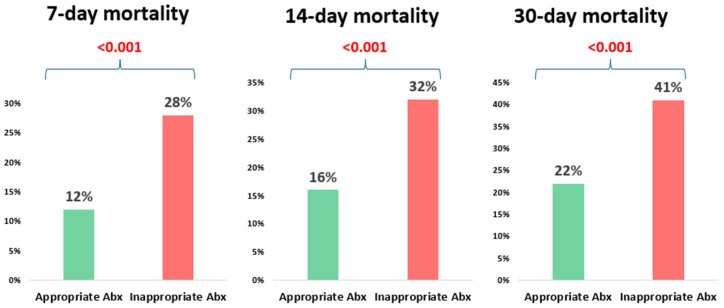
The impact of the appropriateness of empiric antibiotic therapy on mortality at different time points.

The overall 30-day mortality in the study cohort was 26% (295/1132). The mortality was significantly higher among patients who received inappropriate empirical antibiotic therapy compared to those who received appropriate therapy, 41% (96/234) vs. 22% (199/898), *p* < 0.001 (Figure 1, Table 2). Patients who received inappropriate empirical antibiotic therapy also had longer hospital stays compared to those who received appropriate empirical therapy (median 7 days vs. 11 days, *p* < 0.001). There was no significant difference in ICU admission within one week of bacteremia onset, 0% (0/234) vs. 1.3% (12/898), *p* = 0.14 (Table 2).

Table 3 shows the breakdown of the type of empiric antibiotic treatment in both groups. Cephalosporins comprised the majority of the appropriate empiric treatment, 37.6% (338/898), followed by Aminoglycosides, 23% (208/898). Piperacillin-Tazobactam was prescribed equally in both groups, 16.7% (150/898) vs. 15% (35/234), *p* = 0.5. However, Carbapenems were prescribed more in the appropriate empiric treatment group, 18.7% (168/898) vs. 2.5% (6/234), *p* < 0.01.

### 3.4. Risk Factors for 30-Day Mortality

Patients with *Klebsiella* bacteremia who deceased within 30 days were older (median age 73 years vs. 70 years, *p* = 0.002), needed more assistance in ADL, had a higher Charlson comorbidity score (median 6 vs. 5, *p* < 0.001), and had more prior hospitalizations (56% (130/232) vs. 45% (375/837), *p* = 0.002). At bacteremia onset, patients with higher SOFA scores were more likely to die within 30 days (median SOFA score 5 vs. 3 points, *p* < 0.001). In addition, patients who were mechanically ventilated at bacteremia presentation were more likely to die, too, 24% (55/232) vs. 9% (77/837), *p* < 0.001. Regarding laboratory parameters, those with elevated creatinine levels or with lower albumin levels were more likely to die within 30 days [1.55 (0.98–2.62) vs. 1.25 (0.87–1.99), *p* = 0.002 and 2.7 (2.3–3.3) vs. 3.2 (2.8–3.6), *p* < 0.001, respectively] (Table 4). Furthermore, patients who received appropriate empirical antibiotic therapy survived longer during 30 days than those who received inappropriate empirical therapy, 84% (699/837) vs. 72% (168/232), *p* < 0.001. Regarding the source of bacteremia, the mortality rate was higher in patients with an unknown source of 28% (64/232) vs. 23% (190/837) or an abdominal/biliary source, 16% (38/232) vs. 13% (108/837), *p* < 0.001. In contrast, patients with urinary tract-associated bacteremia had a lower mortality rate, 46% (383/837) vs. 29% (67/232), *p* < 0.001 (Table 4). ESBL bacteremia was significantly associated with a higher mortality rate (44% (102/232) vs. 35% (293/837), *p* = 0.01) (Table 4).

**Figure 2:**
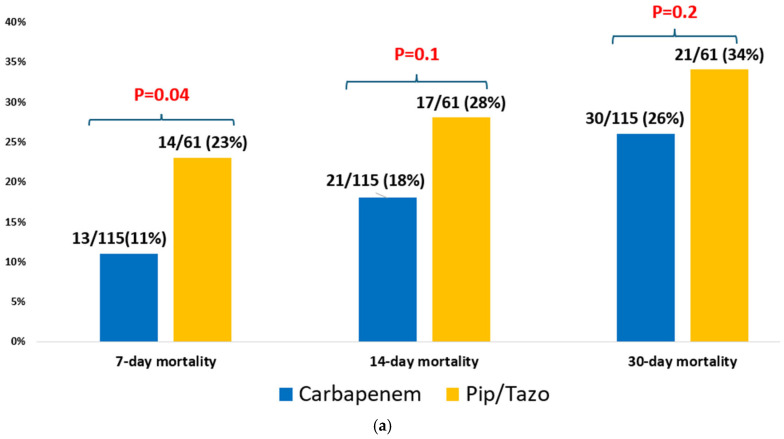

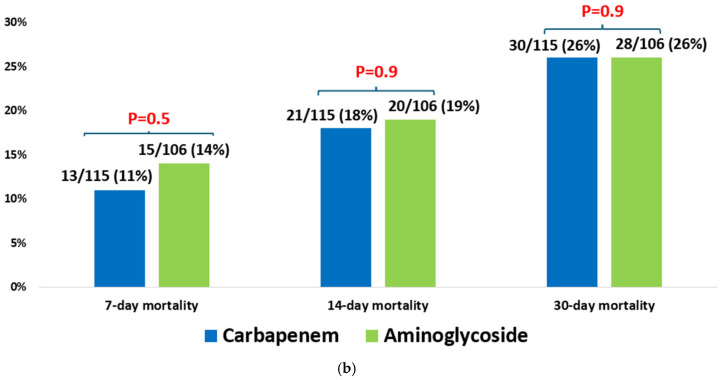
(**a**) Empiric piperacillin-tazobactam vs. carbapenem for ESBL *Klebsiella* spp. bacteremia (*n* = 176). (**b**) Empiric aminoglycoside vs. carbapenem for ESBL *Klebsiella* spp. bacteremia (*n* = 221).

In multivariable analysis, appropriate empirical antibiotic therapy remained significantly associated with reduced 30-day mortality (OR = 0.59, CI95% (0.46–0.76)]. Other risk factors significantly associated with higher mortality included a higher Charlson comorbidity score [OR = 1.06, CI95% (1.02–1.10)], assistance in ADL [OR = 2.16 (1.67–2.79)], prior hospitalization [OR= 1.31 (1.02–1.69)], and a higher SOFA score [OR= 1.32 (1.26–1.38)] (Table 5). We performed additional analyses of patients with ESBL Klebsiella bacteremia who received appropriate empirical therapy and compared different antibiotic groups, assessing their impact on mortality (Figure 2). In one analysis of 167 patients with ESBL *Klebsiella* bacteremia, patients who received empirical carbapenem monotherapy survived more than those who received piperacillin-tazobactam monotherapy, 26% (30/115) vs. 34% (21/61), *p* = 0.2 (Figure 2a). In another analysis of 221 patients with ESBL *Klebsiella* bacteremia, there was no difference between the mortality rate in patients who received empirical aminoglycoside monotherapy and those who received empirical carbapenem monotherapy, 26% (28/106) vs. 26% (30/115), *p* = 0.9 (Figure 2b).

## 4. Discussion

This retrospective study demonstrates that the early administration of appropriate empirical antibiotic therapy significantly reduces 30-day mortality in hospitalized patients with *Klebsiella* bacteremia. These findings are strongly supported by the existing body of literature. The systematic review and meta-analysis by Lodise et al. [10], which focused on *K. pneumoniae* and *E. coli* infections, established that delayed appropriate therapy (>24 h) was associated with a significantly increased risk of mortality (OR = 1.60 [95% CI%, 1.25–2.50]). This body of evidence underscores the critical importance of timely appropriate empirical therapy in *Klebsiella* bacteremia.

The existing literature presents conflicting findings regarding the impact of appropriate empirical antibiotic therapy on mortality in Gram-negative bacteremia. Early retrospective studies from the late 1990s suggested that patients who received inappropriate empirical therapy had significantly higher mortality rates [11,12]. In contrast, more recent studies have reported no significant difference in mortality between patients who received appropriate versus inappropriate empirical therapy.

For example, Scarsi et al. conducted a retrospective study involving 884 patients with Gram-negative bacteremia, 14% of whom received inappropriate therapy. They found no significant difference in mortality between the two groups (OR = 0.61; 95% CI, 0.31–1.18; *p* = 0.14) [13]. Similarly, a prospective multicenter study from England involving 679 patients found no association between inappropriate empiric therapy and 30-day mortality (adjusted OR = 0.92; 95% CI, 0.5–1.66) [1].

On the other hand, a retrospective study from South Carolina, which included 390 patients, found that inappropriate empirical therapy significantly increased mortality, but only in patients with a high baseline risk of death. For patients with mortality risk scores of 5–9 and >10, the adjusted hazard ratios were 3.55 (95% CI, 1.22–9.31) and 4.99 (95% CI, 1.09–22.87), respectively, compared to those with a score <5 (HR = 3.34; 95% CI, 0.17–22.77) [6].

A prospective multicenter study from Spain that included 801 patients with bloodstream infections (71 of whom had *Klebsiella* bacteremia) found that inappropriate empirical therapy was significantly associated with increased mortality (OR = 1.7; 95% CI, 0.98–2.98) [14]. In a more recent retrospective study, appropriate empirical therapy was associated with a significantly lower risk of in-hospital death (OR = 0.52; 95% CI, 0.42–0.64). A sensitivity analysis by pathogen showed that this association held true for patients with *Klebsiella* bacteremia as well (adjusted OR = 0.54; 95% CI, 0.31–0.93). Notably, most patients in this study did not have hospital-acquired infections, and ESBL-producing strains accounted for only 7.2% of Gram-negative bacteremia cases [15].

Our findings also support existing evidence that host clinical factors, such as higher SOFA scores and the presence of invasive devices like central venous catheters and mechanical ventilation at the onset of bacteremia, are independently associated with increased mortality. These results underscore that host condition and severity of illness at presentation remain key determinants of outcomes. Additionally, the emerging literature highlights the role of bacterial characteristics, such as hypervirulence, in predicting mortality. The study by Wu et al. [16] found that the carriage of specific virulence genes was an independent mortality risk factor in *K. pneumonia* bacteremia, suggesting that outcomes are shaped by a complex interplay of host, treatment, and organism factors.

Importantly, our study found no significant difference in mortality between ESBL-producing and non-ESBL Klebsiella bacteremia, suggesting that the appropriateness of empirical therapy may outweigh the specific resistance mechanisms in predicting patient survival. This reinforces the value of timely and targeted empiric treatment regardless of resistance patterns. However, the meta-analysis by Kohler et al. [17] demonstrated significantly higher mortality in patients with carbapenem-resistant K. pneumonia bacteremia compared to carbapenem-sensitive strains, emphasizing that resistance mediates outcomes largely through a lower probability of receiving appropriate initial therapy. This concept is further reinforced by a recent meta-analysis by Maraolo et al., which concluded that carbapenem resistance is independently associated with a worse outcome (aOR 2.17; 95% CI 1.56–3.04), even after adjusting for the activity of the antimicrobial regimen [18].

Although the empirical use of carbapenems was associated with lower mortality compared to piperacillin–tazobactam in our subgroup analysis, the difference did not reach statistical significance (*p* = 0.2). However, this analysis is limited by the small sample size. In addition, similar to the findings of the MERINO trial, the lack of molecular data (e.g., OXA-type beta-lactamases) limits the strength of the conclusion that mortality outcomes were similar between the two groups [7].

Similarly, no significant difference in mortality was observed between patients treated empirically with carbapenems versus aminoglycosides. However, data regarding safety outcomes such as nephrotoxicity and ototoxicity are lacking. These findings suggest that, when guided by local susceptibility patterns, there may be flexibility in empiric treatment choices and offering valuable insights for antibiotic stewardship. However, the limited sample sizes in these subgroup analyses warrant cautious interpretation.

Our study has several limitations. Its retrospective design introduces potential for unmeasured confounding. While we utilized the inverse probability treatment weighted (IPTW) model to mitigate time-dependent confounding and bias, we acknowledge that methods specifically designed to handle competing risks (e.g., Fine and Gray models) may offer additional insights into mortality. Furthermore, while the IPTW analysis was employed to minimize selection bias and balance baseline characteristics, our study lacks formal diagnostic assessments of the weighting process. Specifically, Standardized Mean Differences (SMDs) after weighting, distributions of stabilized weights, and checks for the positivity assumption were not presented. Given the significant baseline imbalances in comorbidities and resistance patterns (e.g., ESBL rates and mechanical ventilation), there remains a risk of residual confounding if perfect balance is not achieved. Additionally, the positivity assumption may have been strained in certain subgroups, such as patients with CPE, where empirical therapy was almost universally inadequate. Consequently, our estimates, including the reported OR of 0.59 for mortality, should be interpreted with caution, as they represent an association within the constraints of this retrospective observational design without formal balance validation.

Regarding the microbiological analysis, molecular typing of ESBL-producing strains and detection of the *ampC* gene were not performed. It is important to note that unlike *Enterobacter* or *Serratia* species, which are known for inducible chromosomal AmpC, *Klebsiella* species typically lack these enzymes, and their AmpC production is predominantly plasmid-mediated. Nevertheless, the lack of molecular characterization limits our ability to distinguish between specific resistance mechanisms or evaluate the risk of selecting for derepressed mutants during therapy.

Furthermore, while our analysis compared clinical outcomes between carbapenems and aminoglycosides, we lacked data on critical safety outcomes, such as the incidence of nephrotoxicity or ototoxicity. This trade-off is a significant consideration in septic cohorts treated with aminoglycosides. Additionally, because our study design specifically excluded patients who received combination empirical treatment to focus on the impact of monotherapy, we cannot draw conclusions regarding the safety or efficacy of combination regimens. Finally, data regarding the exact timing of antibiotic administration and delays in source control were lacking, and the small number of CPE isolates might limit the generalizability of some conclusions to regions with higher endemicity of carbapenem resistance.

Further prospective studies employing advanced causal inference methods are needed to assess the impact of rapid diagnostics on reducing inappropriate empirical therapy.

In conclusion, appropriate empirical therapy plays a key role in improving outcomes among patients with *Klebsiella* bacteremia. By emphasizing the importance of timely and effective treatment, as well as thoughtful antibiotic selection, this study provides valuable insights to guide clinical decision-making and optimize patient care in the context of rising antimicrobial resistance.

## Figures and Tables

**Table 1:** Demographic and clinical characteristics of hospitalized patients with *Klebsiella* spp. bacteremia divided by the appropriateness of empiric antibiotic therapy.

	Entire Cohort (*n* = 1132)	Inappropriate Treatment (*n* = 234)	Appropriate Treatment (*n* = 898)	*p* Value
Age, median (IQR)	71 (60–79)	69 (59–79)	71 (60–80)	0.2
Female gender, *n* (%)	457 (40%)	95 (41%)	362 (40%)	0.9
BMI, median (IQR) (*n* = 961)	25.5 (22.3–29.1)	25.5 (22.5–29.7)	25.5 (22.3–29.1)	0.5
Assistance in ADL, *n* (%)	565 (50%)	119 (51%)	446 (50%)	0.7
Home residency, *n* (%)	740/924 (80%)	132/182 (73%)	608/742 (82%)	0.004
Prior hospitalization in 90 days, *n* (%)	534 (47%)	114 (49%)	420 (47%)	0.6
Surgical ward, *n* (%)	289 (26%)	77 (33%)	212 (24%)	0.004
Charlson score, median (IQR)	5 (4–9)	5 (4–8)	5 (4–9)	0.5
Ischemic heart disease, *n* (%)	198 (17.5)	44 (18.8)	154 (17.1)	0.5
Congestive heart failure, *n* (%)	141 (13%)	49 (21%)	92 (10%)	<0.001
Chronic renal failure, *n* (%)	55 (5%)	14 (6%)	41 (5%)	0.4
Hemodialysis, *n* (%)	55 (4.9)	10 (4.3)	45 (5)	0.6
Diabetes mellitus, *n* (%)	306 (27%)	65 (28%)	241 (27%)	0.8
Solid Tumor, *n* (%)	279 (25%)	45 (19%)	234 (26%)	0.03
Hematological Malignancy, *n* (%)	57 (5%)	16 (7%)	41 (5%)	0.2
*Klebsiella* species				
*K. pneumonia*	992 (87.6%)	213 (91%)	779 (87%)	0.02
*K. oxytoca*	70 (6.2%)	5 (2.1%)	65 (7%)	0.02
*K. aerogenes*	70 (6.2%)	16 (6.8%)	54 (6%)	0.02
Resistance mechanism				
ESBL, *n* (%)	420 (37.1%)	157 (67.1%)	263 (29%)	<0.001
CPE, *n* (%)	41 (3.6%)	37 (15.8%)	4 (0.4%)	<0.001
Bacteremia source	*n* = 1130	*n* = 234	*n* = 896	
Urinary tract, *n* (%)	458 (40.5%)	72 (30.8%)	386 (43%)	0.001
Primary, *n* (%)	291 (25.8%)	70 (29.9%)	221 (25%)	
Abdominal/biliary, *n* (%)	149 (13.2%)	25 (10.7%)	124 (14%)	
Infected wounds, *n* (%)	103 (9.1%)	28 (12%)	75 (8%)	
Indwelling central line, *n* (%)	66 (5.8%)	19 (8.1%)	47 (5%)	
Respiratory tract, *n* (%)	63 (5.6%)	20 (8.5%)	43 (4%)	
Patient characteristics at bacteremia onset				
SOFA score, median (IQR)	4 (2–6)	4 (2–6)	4 (2–6)	0.02
Mechanical ventilation, *n* (%)	147 (13%)	47 (20%)	100 (11%)	<0.001
WBC count, median (IQR) (*n* = 701)	10.1 (6.6–14.9)	9.4 (5.7–15.2)	10.2 (6.6–14.9)	0.6
Creatinine, median (IQR) (*n* = 583)	1.28 (0.91–2.08)	1.47 (0.98–2.52)	1.26 (0.87–2.01)	0.02
Albumin, median (IQR) (*n* = 691)	3.1 (2.6–3.5)	2.95 (2.5–3.5)	3.2 (2.7–3.5)	0.02

IQR—interquartile range, BMI—body mass index, ADL—activity of daily living, ESBL—extended-spectrum beta-lactamase, CPEs—carbapenemase-producing enterobacterales, SOFA—Sequential Organ Failure Assessment, WBC—white blood cell.

**Table 2:** Clinical outcomes in hospitalized patients with *Klebsiella* spp. bacteremia according to the appropriateness of empiric antibiotic therapy.

Variables	Inappropriate Treatment (*n* = 234)	Appropriate Treatment (*n* = 898)	*p* Value
30-day mortality	96 (41%)	199 (22%)	<0.001
14-day mortality	74 (32%)	145 (16%)	<0.001
7-day mortality	65 (28%)	109 (12%)	<0.001
ICU admission a week after culture collection	0 (0%)	12 (1.3%)	0.14
Duration of hospitalization	11 (5–23)	7 (4–14)	<0.001

ICU—intensive care unit.

**Table 3:** Empiric antibiotic therapy type given within 24 h of blood culture collection divided according to the appropriateness of antibiotics.

Empiric Antibiotic Type	Appropriate Antibiotics (*n* = 898)	Inappropriate Antibiotics (*n* = 234)	*p* Value
Cephalosporins, *n* (%)	338 (37.6%)	97 (41%)	0.3
Aminoglycosides, *n* (%)	208 (23%)	5 (2.1%)	<0.01
Piperacillin-Tazobactam, *n* (%)	150 (16.7%)	35(15%)	0.5
Fluoroquinolones, *n* (%)	29 (3.2%)	15 (6.4%)	0.02
Carbapenems, *n* (%)	168 (18.7%)	6 (2.5%)	<0.01
Colistin, *n* (%)	5 (0.5%)	0 (0%)	0.3
No treatment within 24 h, *n* (%)	-	76 (32%)	<0.01

**Table 4:** Univariate analysis for risk factors for 30-day mortality in hospitalized patients with *Klebsiella* spp. bacteremia.

	All Cohort (*n* = 1069)	Survived (*n* = 837)	Deceased (*n* = 232)	*p* Value
Age, median (IQR)	71(60–79)	70 (59–79)	73 (63.2–81)	0.002
Female gender, *n* (%)	424 (40%)	336 (40%)	88 (38%)	0.5
BMI, median (IQR)		25.7 (22.6–29.1)	24.8 (22–29.4)	0.2
Assistance in ADL, *n* (%)	521 (49%)	372 (44%)	149 (64%)	<0.001
Home residency, *n* (%)	704/870 (81%)	561/675 (83%)	143/195 (73%)	0.002
prior hospitalization in 90 days, *n* (%)	505 (47%)	375 (45%)	130 (56%)	0.002
Surgical ward, *n* (%)	283 (27%)	242 (29%)	41 (18%)	<0.001
Charlson comorbidity score, median (IQR)	5 (4–9)	5 (4–8)	6 (4–11)	<0.001
Congestive heart failure, *n* (%)	131 (12%)	86 (10%)	45 (19%)	<0.001
Ischemic heart diseases, *n* (%)	187 (18%)	136 (16%)	51 (22%)	0.04
Diabetes mellitus, *n* (%)	295 (28%)	219 (26%)	76 (33%)	0.05
Chronic renal failure, *n* (%)	53 (5%)	50 (6%)	3 (1.3%)	0.003
Hemodialysis, *n* (%)	54 (5.1)	48 (5.7)	6 (2.6)	0.053
Solid Tumor, *n* (%)	268 (25%)	202 (24%)	66 (28%)	0.2
Hematological Malignancy, *n* (%)	53 (5%)	33 (4%)	20 (9%)	0.004
Characteristics at bacteremia onset				
SOFA score, median (IQR)	4 (2–6)	3 (2–5)	5 (4–7)	<0.001
Mechanical ventilation, *n* (%)	132 (12%)	77 (9%)	55(24%)	<0.001
WBC, median (IQR)	10.1 (6.6–14.9)	9.97 (6.5–14.3)	10.4 (6.6–17)	0.1
Creatinine, median (IQR)	1.28 (0.89–2.1)	1.25 (0.87–1.99)	1.55 (0.98–2.62)	0.002
Albumin, median (IQR)	3.1 (2.6–3.5)	3.2 (2.8–3.6)	2.7 (2.3–3.3)	<0.001
Appropriate empirical treatment, *n* (%)	867 (81%)	699 (84%)	168 (72%)	<0.001
Bacteremia source				
Urinary tract, *n* (%)	450 (42%)	383 (46%)	67 (29%)	<0.001
Indwelling central Line, *n* (%)	64 (6%)	51 (6%)	13 (6%)	
Abdominal/biliary tract, *n* (%)	146 (14%)	108 (13%)	38 (16%)	
Infected wounds, *n* (%)	99 (9%)	71 (9%)	28 (12%)	
Respiratory tract, *n* (%)	55 (5%)	33 (4%)	22 (10%)	
Primary, *n* (%)	254 (24%)	190 (23%)	64 (28%)	
Resistance mechanisms				
ESBL infection, *n* (%)	395 (37%)	293 (35%)	102 (44%)	0.01
CPE infection, *n* (%)	36 (3%)	25 (3%)	11 (5%)	0.2

IQR—interquartile range, BMI—body mass index, ADL—activity of daily living, ESBL—extended-spectrum beta-lactamase, CPEs—carbapenemase-producing enterobacterales, SOFA—Sequential Organ Failure Assessment, WBC—white blood cell.

**Table 5:** Risk factors for 30-day mortality, univariate and multivariate generalized linear models (generalized estimating equation), adjusted by inverse propensity score weighting. N = 1068, AIC = 1898.2, constant β = 2.651.

Risk Factor	Univariate Analysis OR (95% CI)	Multivariate Analysis OR (95% CI)	*p* Value
Appropriate empiric treatment	0.52 (0.37–0.73)	0.59 (0.46–0.76)	<0.001
Older age	1.02 (1.007–1.03)	1.008 (0.99–1.02)	0.3
Prior hospitalization	1.57(1.17–2.10)	1.31 (1.02–1.69)	0.03
Assistance in ADL	2.24 (1.66–3.03)	2.16 (1.67–2.79)	<0.001
High Charlson comorbidity score	1.08 (1.04–1.12)	1.06 (1.02–1.10)	0.03
Hemodialysis	0.43 (0.18–1.03)	0.32 (0.16–0.62)	0.001
Central venous line	1.69 (1.23–2.33)	1.11 (0.84–1.46)	0.4
Mechanical ventilation	3.07 (2.09–4.49)	1.04 (0.73–1.48)	0.8
Higher SOFA score	1.38 (1.29–1.47)	1.32 (1.26–1.38)	<0.001
Urinary source vs. other sources of bacteremia	0.48(0.35–0.66)	0.571 (0.53–0.75)	<0.001
ESBL vs. non-ESBL bacteremia	1.46 (1.08–1.96)	0.92 (0.71–1.18)	0.5

ADL—activity of daily living, ESBL—extended-spectrum beta-lactamase, SOFA—Sequential Organ Failure Assessment.

## Data Availability

The original contributions presented in this study are included in the article.
